# Obesity-promoting factors in Mexican children and adolescents: challenges and opportunities

**DOI:** 10.3402/gha.v9.29625

**Published:** 2016-01-18

**Authors:** Magaly Aceves-Martins, Elisabet Llauradó, Lucia Tarro, Rosa Solà, Montse Giralt

**Affiliations:** 1 Health Education and Promotion, Facultat de Medicina i Ciències de la Salut, Universitat Rovira i Virgili, Reus, Spain; 2 Functional Nutrition, Oxidation and Cardiovascular Disease Research Group, Medicine and Surgery Department, Universitat Rovira i Virgili, Reus, Spain; 3 Hospital Universitari Sant Joan, Centre Tecnològic de Nutrició i Salut, IISPV, Reus, Spain; 4 Unit of Pharmacology, Facultat de Medicina i Ciències de la Salut, Reus, Spain

**Keywords:** health determinants, developing countries, childhood obesity, health and environmental change

## Abstract

**Background:**

Mexico is a developing country with one of the highest youth obesity rates worldwide; >34% of children and adolescents between 5 and 19 years of age are overweight or obese.

**Objectives:**

The current review seeks to compile, describe, and analyze dietary conditions, physical activity, socioeconomic status, and cultural factors that create and exacerbate an obesogenic environment among Mexican youth.

**Design:**

A narrative review was performed using PubMed and the Cochrane Library databases, as well as grey literature data from the Mexican government, academics, and statistical reports from nongovernmental organizations, included in electronic formats.

**Results:**

The recent socioeconomic and nutritional transition has resulted in reduced healthy meal options at public schools, high rates of sedentary lifestyles among adolescents, lack of open spaces and playgrounds, socioeconomic deprivation, false or misunderstood sociocultural traditional beliefs, misconceptions about health, a high percentage of overweight or obese adults, and low rates of maternal breastfeeding. Some of the factors identified are exacerbating the obesity problem in this population. Current evidence also shows that more policies and health programs are needed for prevention of childhood and adolescent obesity. Mexico presents alarming obesity levels, which need to be curtailed and urgently reversed.

**Conclusions:**

The present narrative review presents an overview of dietary, physical activity, societal and cultural preconceptions that are potentially modifiable obesity-promoting factors in Mexican youth. Measures to control these factors need to be implemented in all similar developing countries by governments, policy makers, stakeholders, and health care professionals to tackle obesity in children and young people.

## Introduction

According to the World Health Organization (WHO), *obesity* is defined as the fatness level sufficient to increase risk of morbidity or mortality (1). The prevalence of this health issue in young populations has risen substantially worldwide in less than one generation, especially in developing countries (2).

Excess body fat in children and adolescents can lead to a variety of clinical conditions and psychosocial disorders such as nonalcoholic fatty liver disease, sleep apnea, type 2 diabetes, asthma, cardiovascular disease, hypercholesterolemia, glucose intolerance and insulin resistance, skin conditions, menstrual abnormalities, impaired balance, orthopedic problems, and poor learning skills, which can lead to social discrimination. All of these conditions contribute to an increased morbidity and/or premature mortality (2–6). The worldwide prevalence of overweight and obesity in children under 5 years of age increased from 4.2% in 1990 to 6.7% in 2010. This trend is expected to continue rising to reach an estimated prevalence of 9.1% in 2020 (7). In low-to-middle-income countries of Latin America there have been rising rates of overweight and obesity over the past three decades, which have not spared children and adolescents (7, 8). It is estimated that one of every four or five children and adolescents in Latin America is overweight or obese, which represents 20–25% of the overall youth population in this region (9, 10).

Mexico is one of the developing Latin American countries and is the 11th most populated in the world (11). Almost half of the population is classified as poor or vulnerable to social deprivation (12, 13). As with many countries with similar characteristics, during the last 30 years Mexico has suffered from several demographic, economic, environmental, and cultural changes that have had an adverse impact on its citizens’ lifestyle and well-being. Approximately 75% of all deaths in Mexico are caused by noncommunicable diseases (14). Obesity and unhealthy diets are among the main risk factors of mortality (14, 15), and obesity-related comorbidities end the lives of 28% of Mexicans per year, a total of 170,000 people (16).

Obesity rates in Mexico have reached near-epidemic proportions, and generalized obesity appeared before the earlier historic malnutrition problem was solved. Therefore, both conditions currently coexist (13, 17). These two nutritional problems within the same population represent a high risk of obesity for the most vulnerable sectors – adults living in poverty and their offspring (13, 17–20).

Childhood obesity is a problem that is increasing rapidly in Mexico (21). According to obesity prevalence estimates, using WHO reference data, for children younger than 5 years of age Chile has the highest prevalence of obesity at 10.3% (2012) (22), followed by 9.8% in Mexico (2012), 6.9% in Peru (2007–2010), and 5.2% in Colombia (2010). For school-aged children, Mexico tops the list at 11.8% for girls and 17.4% for boys (2012), followed by 14.3% in Brazil (2009) and 5.2% in Colombia (2010). In adolescent populations, the data suggest that Mexico has the highest rates, with 12.1% for girls and 14.5% for boys (2012) followed by 7.3% in Brazil (2009) and 3.4% in Colombia (2010) (10).

Once established, obesity is difficult to treat, and the excess body weight in youth increases the risk of presenting obesity in adulthood; with the concomitant increased risk of early-onset obesity and the related noncommunicable diseases (23–25).

Childhood obesity is the result of complex and interacting dynamics that create an obesogenic environment, which includes biological, social, and behavioral factors acting within the influence of the youth's family and community environment, in which the available options tend towards exacerbating the problem (3–6). However, although young Mexicans grow up in a more obesogenic environment than other cultures, not all these children and adolescents become obese. Thus, understanding obesity-promoting factors, how these factors interact with the youth environment, and how these interactions predispose some youngsters to becoming overweight or obese are important matters for future preventive approaches.

Scientific information is scarce regarding these factors in Mexican children and adolescents. Thus, the aim of the current narrative review is to compile, describe, and analyze dietary conditions, physical activity, socioeconomic status, and cultural factors that create and exacerbate an obesogenic environment among Mexican young people – factors that need to be considered for intervention, health programs, and policies in the near future.

## Mexican youth obesity rates

Between 1999 and 2012, the prevalence of overweight and obesity increased among 5- to 11-year-olds from 28.2 to 36.9% (0.7 percentage points/year) in boys, and from 25.5 to 32.0% (0.5 percentage points/year) in girls. Between 1988 and 2012, obesity prevalence among 12–19 year-old adolescents increased from 11.1 to 35.8% (1.0 percentage points/year) in females, while in males it went from 33.0 to 34.1% (13). This increase was established between 2006 (when obesity was defined in Mexico) and 2012 (the latest obesity data available) and, consequently, the calculated increase is low (0.2 percentage points/year) (13). Likewise, the most recent data available from the 2012 Mexican National Survey of Health and Nutrition (26) describes the prevalence of obesity and overweight status in Mexican children and adolescents (Table 1).

**Table 1:** Prevalence of obesity and overweight in Mexican children and adolescents

Population	Prevalence of overweight and obesity (%)	Prevalence of overweight (%)	Prevalence of obesity (%)
5 to 11 year–olds	34.4	19.8	14.6
Girls	32	20.2	11.8
Boys	36.9	19.5	17.4
12 to 19 year-olds	34.9	21.6	13.3
Females	35.8	23.7	12.1
Males	34.1	19.6	14.5

Data obtained from the 2012 Mexican Health and Nutrition National Survey (Encuesta Nacional de Salud y Nutrición).

## Methods

### Obesity-promoting factors in Mexican youth populations

PubMed and the Cochrane Library databases were searched for Spanish or English language manuscripts of obesity-promoting factors in Mexican children and adolescents, using a combination of the connectors ‘AND’ or ‘OR’. The keywords were ‘obesity’, ‘Mexico’, ‘children’, ‘adolescents’, ‘obesogenic environment’, ‘obesity promoter factors’, and ‘developing countries’. The time restraints were publications between January 1990 and March 2015 so as to focus on the contemporary epidemiological and environmental circumstances of childhood and adolescent obesity. However, only a small number of publications relating to Mexican youth populations with the above-mentioned characteristics were found. In some of the publications identified, the obesity-promoting factors in youth were not presented or were referenced to other studies or reports. Hence, we decided to widen the search to include grey literature data from the Mexican government, academics, and statistical reports from nongovernmental organizations (NGOs), including in electronic formats.

A systematic review was not feasible and a narrative review was performed instead. Further, apart from answering specific research questions, the principal aim of this work was to compile, describe, and analyze the obesity-promoting factors that can be identified in Mexican youth. From identified references, every obesity-promoting factor identified in this population, as well as those recognized in studies performed in other developing countries that have very similar population characteristics to Mexico, were collated and discussed. Four main categories were created to organize the information collected: 1) dietetic; 2) physical activity; 3) social, economic, and cultural conditions; and 4) genetic, hormonal, and behavioral alterations. The factors included in each of these categories were classified according to population characteristics: 1) factors identified in the Mexican population that promote obesity among youth; 2) factors in the Mexican population that could promote obesity among youth; and 3) factors identified in developing countries that could promote obesity among Mexican youth. Further, the types of studies included in this narrative review were identified.

## Results

### Potential modifiable obesity-promoting factors in Mexican youth populations

The following obesity-promoting factors were identified in the youth populations of Mexico or other developing countries.

#### Dietetic

Mexico is suffering through a nutritional transition in which there has been a decline in traditional and homemade diets, contrasting with an increase in the consumption of commercial products, fast food, and food prepared away from home (27, 28). The average Mexican diet is based on high saturated fat, low protein, and low fiber intake (28). Between 1999 and 2006, an increase of 226% in consumption of carbonated drinks and sweetened beverages was documented among Mexican children and adolescents (13). Mexican children have an overall daily water intake below the recommended levels, and the major liquid contributors to total energy intake are high-fat milk, fruit juices, and carbonated and noncarbonated sweetened soft drinks (29–31). Estimates indicate that plain water accounts for only 26.5% of their daily water intake (29). In the school environment, where children and adolescents spend most of their time, healthy food options are nonexistent, and low quality meals have been noted in full-day public schools. A lack of available potable water has coincided with the increased proliferation of calorically enhanced (sweetened) beverages in schools (29, 31). Moreover, a low level of knowledge regarding dietary components in school children has been identified (28).

It is important to note that, even though the number of cases of undernutrition resulting from poverty in children have decreased (32), this condition still exists among children less than 5 years of age, where 2.8% present low weight, 13.6% present low height for age, and 1.6% present emaciation (26). Birth weight is inversely associated with later obesity prevalence in children (33). Dietary behavior is one part of the primary comportments influencing energy balance, and it has been used in childhood and adolescent treatments against obesity (33).

#### Physical activity

The 2012 Health and Nutrition National Survey (Encuesta Nacional de Salud y Nutrición-ENSANUT 2012) showed that 58.6% of children between the ages of 10 and 14 reported doing no extracurricular physical activity, whereas 67% spent more than 2 h/day in front of a television screen, a computer screen, and/or a gaming console (26). In the last decades, a shift in activity patterns has been noted, including increased indoor entertainment and decreased outdoor activities (34).

Within the school environment, three salient factors are of note: only 1 h/week of physical education is mandatory in Mexican schools; 96% of the teachers in charge of physical education do not program their classes; and there is a lack of open spaces and playgrounds in schools and communities for performing physical activity (35–37). In addition, there is increasing pressure on children to perform academically, with reduced emphasis on sports (3). Physical activity and sedentary behavior are another part of the primary factors that influence energy balance and that have been used in childhood and adolescent obesity treatments (24).

Other factors that promote sedentary behavior are the existence of a large number of insecure and unsafe neighborhoods, which discourages children from staying outdoors and playing with their friends (36). Time spent outdoors is one of the strongest correlates of physical activity among children, whereas the lack of security in playgrounds is one of the primary factors that parents consider when selecting locations in which to allow their children free-time physical activity (38). Additionally, there are few open spaces and playgrounds in schools and communities, which results in reduced physical activity. Finally, beliefs that girls should not play sports, but should rather be mostly engaged in household chores, still prevail in Mexican society (3, 37).

#### Social, economic, and cultural conditions

Statistical analyses relating globalization to increasing obesity rates are difficult to quantify (39). Nevertheless, globalization may contribute to nutritional transition in developing countries by being simultaneously a product and a driver of technological change, thus providing similar causal links with overweight and obesity (3, 39).

In Mexico, noticeable socioeconomic deprivation has been described, involving high poverty levels where 45.5% of the population is classified as poor and 28.6% as vulnerable to social deprivation (12, 40, 41). Low household incomes, where the main income is about US $5/day for the average family of four members (40, 41), exacerbate the consequences of healthier diets being more expensive than other (cheaper) diets (42–46).

The nearly 2,000-mile border with the United States and friendly trade relations between the countries make it easier to pump junk food into Mexico. After the United States, Canada, and Mexico signed the North American Free Trade Agreement in 1994, processed food sales grew by 5–10% per year between 1995 and 2003 (22).

Since 1990, Mexico has experienced an important rural-to-urban migration (47). According to the National Statistics and Geography Institute (41), a *rural area* is defined as a region where fewer than 2,500 inhabitants live, whereas *urban areas* have more than 2,500 inhabitants. Nowadays, more than 76% of the population lives in urban areas, leaving less than 24% in rural regions (44). Rural-to-urban migration and rapid urban growth may contribute to the shift in the diet of rural migrants, who abandon diets that are typically rich in vegetables and cereals in favor of those high in processed meat and animal fat content. This change of diet is accompanied by reduced levels of physical activity, resulting in increased rates of overweight and obesity (48–50).

Traditional mistaken beliefs and misunderstandings about health and nutrition persist down the generations, where many mothers and grandmothers still believe that overweight or obese children have the appearance of health, leading to overprotection and overfeeding by parents or grandparents (22–36).

High exposure to TV advertising of food items has been identified as a contributing factor (45). On average, 61 TV commercials per day are transmitted, of which 42% are related to the consumption of food items that are conducive to obesity (37). Added to this, these products can be easily acquired outside schools, cinemas, theatres, and recreational sites. As such, obesity among youth is actively promoted by social factors of peer pressure that are exploited by advertising (37).

#### Genetic, hormonal, and behavioral alterations

Genetic and hormonal factors are also promoter agents within the population, but they are less predominant than lifestyles and habits (3).

It is important to note the observations of Lobstein et al. (51), who showed that Mexico's data follow a pattern where children's heights are consistently below the WHO reference values (by about 6 cm) and their weights are also lower than reference values (by about 1.4 kg). In addition, their body mass index (BMI) values are consistently higher than WHO reference standards (about 1.1 kg/m^2^ above). Therefore, an apparent increase in the prevalence of overweight cannot be attributed entirely to excess bodyweight *per se*, but is a value that is confounded by a low height for age in these children (51).

Mexican children have also reported altered sleep patterns, which are also related to higher BMI in childhood and adolescence (52, 53). Furthermore, approximately 73% of Mexican adult females and 69.4% of Mexican adult males are overweight or obese (26), and as parents they play an important role in the quantity and quality of food consumed and the activity patterns of their children. This implies transference of eating and lifestyle patterns from parents to children (54, 55). Of the women of childbearing potential, 73% are overweight or obese, and only 14.4% of Mexican mothers breastfeed their children through the first 6 months of life (26). Maternal pregestational overweight or obesity is negatively associated with breastfeeding incidence and duration and positively associated with the child's weight (56, 57). The impact of pregestational overweight is a threefold higher risk of childhood obesity, whereas excessive gestational weight gain is associated with a 33% offspring obesity (56, 57). Moreover, children who are breastfed have an approximate 24% less risk of presenting overweight or obesity (57).

Data on Mexican dietary constituents and physical activity practice patterns, together with social, economic, cultural, behavioral, hormonal, and genetic alterations that promote obesity among children and adolescents, are summarized in Table 2.

**Table 2:** Factors that could promote obesity in Mexican children and adolescents

Factor category	Population	Factors	Type of study/reference
Dietetic	Factors identified in the Mexican population that promote obesity among youth	Lack of healthy beverage options at schools, as well as potable plain water at school and home (28–30) Low water consumption and high consumption of carbonated drinks and sweetened beverages (28–30) Cheap and readily available low-priced energy-dense foods (44)	Cross-sectional study (29) Cross-sectional study (30) Cross-sectional study (31) Qualitative phenomenological study (45)
	Factors in the Mexican population that could promote obesity among youth	Nutritional transition suffered by the country (26, 27) Diet based on high saturated fat, low protein, and low fiber intake (27) Lack of healthy snacks at the school cafeteria (28) Low level of knowledge regarding dietary components in schoolchildren (26, 27) Low birth weight in Mexican children (25) Undernutrition in Mexican children under 5 years of age (25)	National survey (26) Grey literature^a^ (27) Review (28) Cross-sectional study (29)
	Factors identified in developing countries that could promote obesity among Mexican youth	Lack of healthy options at schools and low quality of school meals (3) Low birth weight in children (32)	Review (3) Cross-sectional study (33)
Physical activity	Factors identified in the Mexican population that promote obesity among youth	Shift in activity patterns from outdoor play to indoor entertainment (33)	Cross-sectional study (34)
	Factors in the Mexican population that could promote obesity among youth	High rates of sedentary lifestyles (25) Insecure and unsafe neighborhoods where children are discouraged from staying outdoors and playing (37) Very few hours devoted to physical education at school (34–36) Classes not programmed by the teachers of physical education in schools (34–36) Lack of open spaces and playgrounds in schools and communities (34–36)	National survey (26) Grey literature^a^ (35) Review (36) Review (37)
	Factors identified in developing countries that could promote obesity among Mexican youth	Pressure on children to perform academically, reduced emphasis on sports (3)	Review (3)
Social, cultural, economic conditions	Factors identified in the Mexican population that promote obesity among youth	Increased exposure of children to advertisements for industrialized, processed foods (45)	Qualitative phenomenological study (45)
	Factors in the Mexican population that could promote obesity among youth	Socioeconomic deprivation (12, 27, 40, 41) High poverty levels (12, 27, 40, 41) Low household incomes (40, 41)	Grey literature^a^ (12) Grey literature^a^ (21) Grey literature^a^ (27)
		Rural to urban migration (27, 33, 47–50) False or misunderstood sociocultural traditional beliefs and misconceptions about health (21, 37) Increase in industrial purchasing power leading to lower processed-food costs (43) Overprotection and overfeeding by parents (21) Foods that are conducive to obesity are easily accessed outside schools, cinemas, theatres, and recreational places (37)	Cross-sectional studies (33) Review (37) Grey literature^a^ (40) Grey literature^a^ (41) National survey (43) Grey literature^a^ (47) National representative surveys (48) Review (49) Review (50)
	Factors identified in developing countries that could promote obesity among Mexican youth	Increase in Westernized lifestyles (3) Globalization and industrialization (39)	Review (3) Review (39)
Other genetic, hormonal, behavioral alterations	Factors in the Mexican population that could promote obesity among youth	Low rates of maternal breastfeeding (26) High percentage of overweight or obese parents (26) Food consumption patterns transferred from parents to children (36) Women with childbearing potential (20–45 years of age) are overweight or obese (36)	National survey (26) Review (36)
	Factors identified in developing countries that could promote obesity among Mexican youth	Less frequent than lifestyle factors are genetic and hormonal disorders predisposing to obesity (3) Bad sleep patterns in children and adolescents (52, 53)	Review (3) Review (52) Cross-sectional study (53)

a Grey literature references include the following: reports (technical reports, statistical reports, market research reports, etc.), bibliographies, technical and commercial documentation and governmental and official documents not published commercially, and newspaper and trusted media network information.

In the present narrative review, the studies included were observational, with five cross-sectional studies, one qualitative phenomenological study, and one quali-quantitative study pertaining to Mexican youth obesity–promoting factors. There were no longitudinal studies (Table 3).

**Table 3:** Types of references identified regarding obesity-promoting factors in Mexican young people

Reference type	Mexican population	Reference number
Mexican Health and Nutrition National Survey (ENSANUT)	ENSANUT data, latest version	26
Cross-sectional studies	ENSANUT data 2,867 children and adolescents (1–18 years old)	29
	ENSANUT data 17,215 children and adolescents	30
	ENSANUT data 1999 416 adolescents (12–18 years old) and 2,180 adults (>19 years old) ENSANUT data 2006 7,464 adolescents (12–18 years old) and 21,113 adults (>19 years old)	31
	ENSANUT data 1988, 1999, 2006, 2012	32
	712 Mexican children (9–16 years old)	34
Qualitative phenomenological studies	53 children (9–10 years old)	45
Quali-quantitative studies	84 low-income, Mexican-origin mothers of children 4 to 6 years old	55
Grey literature and governmental references	–	27, 35, 40, 41, 47
Reviews about Mexican obesity problem	–	28, 36, 37, 46, 61
Support articles (not Mexican population)	–	33, 38, 39, 42, 43, 44, 48, 49, 50,51, 52, 53, 54, 56, 57, 58, 62, 64

### Current strategies performed to reduce children and adolescent obesity in Mexico

The Mexican government and health institutions have implemented several strategies to prevent childhood and adolescent obesity. Unfortunately, most of these actions have not been publicly evaluated as yet, nor have results indicating their effectiveness been published. To achieve the full potential of these interventions, particularly in low- and middle-income countries, evaluations need to be conducted to document the programs’ implementation, and the results should be widely disseminated (58).

One of the latest interventions by the Mexican government was the introduction of taxes on sugar-sweetened beverages of about 1 peso (US $0.06) per liter. Preliminary data published by the Public Health National Institute reports an average decline of 6% in purchases of these taxed beverages and an almost 4% increase in purchases of untaxed beverages in 2014, compared to pretax trends (59). In addition, the international 5-a-Day program has been implemented as a cooperative venture between private and public associations in Mexico. For a qualitative study published on the program, the general population was interviewed and the results showed that none of the persons interviewed considered the program to have a sufficient budget or marketing strategy (60). Moreover, the people interviewed felt that this program was not popular among the Mexican population, and that it lacked the support of the national authorities (60)

In 2015, the Mexican Institute for Competitiveness analyzed the implementation of ongoing federal policies for reducing obesity rates in the country (61). The NOURISHING framework, created by the World Cancer Research Fund for reporting and categorizing comprehensive food policies to promote healthy diets, was used (62). This framework is based on the understanding that food policies to prevent obesity should be aimed at improving dietary behavior by encouraging the availability, affordability, and acceptability of healthy diets (61, 62). The results of this analysis are shown in Table 4.

**Table 4:** Current Mexican national policies for obesity prevention analyzed using the NOURISHING framework

Domain	First letter of acronym	Policy area	Mexico's policies for preventing obesity	IMCO's evaluation
Food environment	N	Nutrition label standards and regulations on the use of claims and implied claims on foods	Mexico has rules about listing the contents of drinks and prepackaged foods – in particular, the front and distinctive nutritional labeling law, modified in 2014.	Policy is appropriate but insufficient to cover this area. The design of this labeling is not self-explanatory and the reference values need to be homogenized. In addition, the rule governing claims or suggestive advertisements for products does not follow the same nutritional criteria.
	O	Offer healthy foods and set standards in public institutions and other specific settings	In 2015, the program to address the infrastructure deficit of safe drinking water to replace sugary drinks in schools will commence. In addition, guidelines for the sale and distribution of food in schools will be implemented.	Policy is appropriate but insufficient to cover this area. The budget for this program is insufficient to cope with the backlog. There is a lack of training of the educational staff and general mechanisms to ensure compliance with this program need to be strengthened. In addition, there are no similar standards for other public institutions such as hospitals and sports centers.
	U	Use economic tools to address food affordability and purchase incentives	In Mexico, since 2014, there has been a special tax on sugary drinks and foods high in calories (275 kcal or more per 100 grams).	Policy is appropriate but insufficient to cover the area. There are no additional tools that encourage consumption of nutritious foods (such as fruits and vegetables) and drinking water.
	R	Restrict food advertising and other forms of commercial promotion	Since 2014, in Mexico there have been guidelines to be followed by food and beverage advertisers that prohibit publicizing foods that exceed nutritional criteria on television and cinema for children's schedules.	Policy is appropriate but insufficient to cover the area. These guidelines are not applied to other media to which children are exposed.
	I	Improve the quality of the food supply	Liconsa (a Mexican milk producer that distributes milk at subsidized prices) has been encouraged to sell low-fat milk since 2011. Further, in 2012, the baking industry has employed a voluntary agreement to reduce sodium in their products.	There are inadequate policies to cover this area There are no agreements or regulations in place to reduce sugar or fat (especially trans fats) in processed foods.
	S	Set incentives and rules to create a healthy retail environment	Only some local examples are identified. Since 2013, the government of Mexico City launched a voluntary initiative for local restaurants to remove the salt dispenser from the customer's table. In addition, by law all restaurants in Mexico City must provide free potable water to customers.	No national policies have been created to cover this area.
Food system	H	Harness supply chain and actions across sectors to ensure coherence with health issues	No implemented policies that cover this area were identified.	No national policies to cover this area have been created.
Behavior change communication	I	Inform people about food and nutrition through public awareness	Mexico has its *Chécate*, *Mídete*, *Muévete* (Check up, measure, and move yourself) campaign for the whole population, launched by the health ministry.	Policy is appropriate but insufficient to cover the area. No warning about the risks associated with unhealthy lifestyles or the benefits that would accrue from changing habits.
	N	Nutrition advice and counselling in health care settings	The Clinical Practice Guidelines (CPG) of 2014 include prevention, diagnosis, and treatment of overweight and obesity in children and adults.	Policy is appropriate but insufficient to cover the area. No training programs for health professionals were identified.
	G	Provide nutrition education and skills	The social development ministry has a training program for community instructors and supervisors, based on desirable food habits.	Policy is appropriate but insufficient to cover the area. Campaigns aimed at other locations, especially at the educational sector, are needed.

Mexican Institute of Competitiveness [Instituto Mexicano para la Competitividad A.C. - IMCO (61)] analysis of ongoing national policies for diminishing obesity rates.

## Discussion

### Challenges and opportunities

After considering all the obesity-promoting factors addressed in this manuscript, it is no surprise that the treatment and prevention of childhood and adolescent overweight and obesity remains a challenge in Mexico. It is clear that the average Mexican youth is surrounded by different factors that can lead to obesity. From a life-course perspective, the concern is that this health issue at this vulnerable period of life will have an impact on health not only at this stage but also in adulthood. Further, the general obesity problem in Mexico is not only a public health issue but also a public financial problem that affects families’ income, reduces the country's competitiveness and productivity, and generates costs of up to 82% of the projected overall health expenditure (61, 63, 64). Consequently, the economic and psychosocial costs of obesity, in combination with the associated comorbidities and sequelae, are outstanding and need to be addressed (58, 61, 62). Further, it is important to note that the majority of the obesity-promoting factors are potentially modifiable through interventions inducing behavioral changes. In the present narrative review, the seven studies included are observational and cross-sectional, but no longitudinal studies were included; hence we cannot evaluate the cumulative effects of chronicity.

From a national policy perspective, the NOURISHING framework opens new avenues that could help monitor the domains and areas that policy makers should cover within an appropriate policy design that helps fight obesity (62). Childhood and adolescence have been increasingly recognized as targetable by policies and interventions to reduce the obesity problem, since there are behavioral and obesity-promoting factors that can be changed at this life stage (55, 65). Additionally, a recent publication that analyzed food policies for the obesity problem concluded that policies should target the food preferences of the young, since comprehensive policy actions could create an enabling environment for youth to learn healthy food preferences and, as well, could target actions that empower disadvantaged populations to overcome barriers in order to acquire healthy preferences (66).

Nevertheless, interventions, health programs, and policies that encourage behavioral change need to include recognition of the influence of the wider social and economic environment (67). They should be adapted to each culture, so that possible barriers can be identified and overcome in order to produce effective health strategies to reduce youth obesity (51).

Multiple strategies that focus on healthy meals, sports, outdoor and home activities, family involvement, school activities, and community participation need to be encouraged (68, 69). Obesity prevention interventions should include the youngsters, parents, health care professionals, policy makers, schools, and the community in general, to ensure a significant effectiveness and impact on obesity prevalence and its long-term reduction (6, 58). Evaluating and disseminating the outcomes of such interventions is also important (6, 58).

Obesity is the result of increased energy intake or decreased energy expenditure, or a combination of both factors. From the results presented in this narrative review, some factors can play a major role in this energy imbalance, for example low water consumption and high consumption of carbonated drinks and sweetened beverages (29–31), in addition to cheap and readily available low-priced energy-dense foods (45). Also important to take into account is the phenomenon of overfeeding by parents (21), which can influence the physiologic mechanisms involved in obesity. These mechanisms have been focused on body-weight set-points based on a detection system in adipose tissue that reflects stored fat and a receptor, located in the hypothalamic centers (70). When fat stores are depleted, the adiposity signals are low, and the hypothalamus responds by stimulating hunger and decreasing energy expenditure to conserve energy (70). Conversely, when fat stores are abundant, the signal is increased, and the hypothalamus responds by decreasing hunger and increasing energy expenditure (70). The recent identification of the *ob* gene (obesity) and its product leptin (71) and the *db* gene, whose product is the leptin receptor (72), provides important elements of a molecular basis for this physiologic concept.

Based on obesity-promoting factors identified in this review, we can propose (Table 5) that childhood and youth obesity prevention in Mexico needs to be a joint effort between Mexican families, stakeholders, and policy makers. The table suggests some recommendations to tackle childhood and youth obesity in Mexican population based on the obesity-promoting factors identified in the literature. The recommendations are aimed at Mexican families and governmental policy makers and stakeholders. Priority recommendations are those combinations of factors identified in Mexican populations that promote obesity among youth. Two priority recommendations were identified for Mexican families: to encourage plain water consumption instead of carbonated and sweetened beverages in children; and to encourage physical activities and sports (indoor and outdoor) in children. Similarly, four priority recommendations were identified for the Mexican government, policy makers, and stakeholders: to focus the food industry, stakeholders, and governmental actions across sectors to ensure coherence in policies aimed at obesity prevention; to set fair prices for fresh fruits and vegetables; to provide the proper infrastructure to supply potable water at schools and public places; and to regulate high caloric content food advertising in cinemas, theaters, public places, and TV programming for children.

**Table 5:** Recommendations to tackle obesity based on obesity-promoting factors identified in the literature

Mexican families	Governmental authorities, policy makers, and stakeholders
Recommendations based on factors identified in Mexican populations that promote obesity among youth Encourage plain water consumption instead of carbonated drinks and sweetened beverages Encourage indoor and/or outdoor physical activities and sports	Recommendations based on factors identified in Mexican populations that promote obesity among youth Encourage the food industry, stakeholders, and governmental actions across sectors to ensure coherence in policies aimed at obesity prevention Set fair prices for fresh fruits and vegetables Provide the proper infrastructure to ensure supply of potable water in schools and public places Regulate high caloric content food advertising in cinemas, theaters, public places, and TV scheduling for children
Recommendations based on factors in Mexican populations that could promote obesity among youth Increase homemade food consumption Return to traditional meals (rich in fruits and vegetables) Discourage outdated sociocultural beliefs against sports and foods Encourage the practice of extracurricular physical activity for boys and girls Guarantee maternal breastfeeding for at least the first 6 months of newborns’ lives Parents should have a healthy weight so they can transfer, by example, healthy lifestyle patterns to their offspring Avoid overprotection and overfeeding of children by parents or other relatives Limit exposure to TV, computer screens, and gaming consoles	Recommendations based on factors in Mexican populations that could promote obesity among youth Increase variety of healthy foods and snacks at the school's cafeteria Increase public security and decrease social disparities Set national initiatives and rules that favor a healthy environment for children and adolescents Set appropriate taxes for foods with high calorie content Incorporate nutrition and healthy habits and skills into school curricula Improve physical education at schools: increase mandatory hours, classes, plans, and playground spaces inside the school's facilities Control high caloric content food access outside schools, cinemas, theaters, and public places Eradicate undernutrition in children Increase household general incomes to ensure the basic household's food basket Promote prenatal monitoring among women to reduce low birth weight infants Establish obesity and overweight preventive programs focused on childbearing women
Recommendations based on factors identified in developing countries that could promote obesity among Mexican youth Encourage extracurricular physical activity or sports activities in children Promote traditional meals and the consumption of organic, low-carbon footprint foods Establish healthy sleep patterns in children and youth Ensure appropriate sleeping hours	Recommendations based on factors identified in developing countries that could promote obesity among Mexican youth Promote prenatal monitoring among women to reduce low birth weight infants Promote healthy, quality food options in school meals Implement nutrition education in schools to increase the consumption of fruits and vegetables Other recommendations Train health care professionals in the prevention, diagnosis, and treatment of overweight and obesity in children and adolescents Alert parents and children to the health problems that obesity can involve Improve the quality of subsidized foods and food supplies to schools Ensure that interventions aimed at reducing obesity have public exposure

## Conclusions

The present narrative review presents an overview of dietary, physical activity, societal and cultural preconceptions that are potentially modifiable obesity-promoting factors in Mexican youth. Measures to control these factors need to be implemented in all similar developing countries by governments, policy makers, stakeholders, and health care professionals to tackle obesity in children and young people.

## Paper context

Mexico is a developing country with one of the highest youth obesity rates worldwide. Obesity is considered a severe health problem, especially in youth. Children and adolescents are a target population for policy making and interventions focused on tackling obesity. However, understanding Mexican youths’ obesogenic environment, obesity-promoting factors, their interactions, and how this could predispose children and adolescents to become overweight or obese is a key matter for future preventive approaches.

## Authors' contributions

MAM, EL, LT, RS, and MG all made substantial contributions to the conception or design, acquisition, analysis, and/or interpretation of data for the present work; each of the authors drafted the work or revised it critically for important intellectual content, and approved this version to be published. All of the authors agreed with all of the aspects presented in this work, ensuring that questions related to the accuracy or integrity of any part of the work were appropriately investigated and resolved.
